# Effects of pain neuroscience education in hospitalized patients with high tibial osteotomy: a quasi-experimental study using propensity score matching

**DOI:** 10.1186/s12891-019-2913-5

**Published:** 2019-11-07

**Authors:** Naoki Deguchi, Yoshiyuki Hirakawa, Shota Izawa, Kazuhito Yokoyama, Keito Muraki, Ryouiti Oshibuti, Yasuki Higaki

**Affiliations:** 1 Fukuoka Reha Orthopedics Clinic, 7-220 Nokata, Nishi-ku, Fukuoka-shi, Fukuoka, 819-8551 Japan; 2 Graduate School of Sports and Health Science, Fukuoka University, Fukuoka, Japan; 3 Department of Rehabilitation, Fukuoka Rehabilitation Hospital, Fukuoka, Japan; 4 Faculty of Sports and Health Science, Fukuoka University, Fukuoka, Japan; 5 Fukuoka University Institute for Physical Activity, Fukuoka University, Fukuoka, Japan

**Keywords:** Knee osteoarthritis, Physical therapist, Propensity score matching, Education, Catastrophizing

## Abstract

**Background:**

Pain neuroscience education (PNE) has been shown to reduce pain or psychological symptoms in patients with chronic pain and preoperative knee osteoarthritis; however, the evidence of its effectiveness in hospitalized patients who have undergone high tibial osteotomy (HTO) is unknown. This study was performed to determine whether the implementation of a newly developed hospital-time PNE provided by physical therapists to patients after HTO can result in meaningful improvements.

**Methods:**

In total, 119 patients aged ≥45 years with knee osteoarthritis who were scheduled to undergo HTO were analyzed. Patients with a low Pain Catastrophizing Scale (PCS) score of < 21 were excluded. The patients were classified into two groups: those who underwent a combination of PNE and rehabilitation (intervention group, *n* = 67) and those who underwent rehabilitation only (control group, *n* = 52). The patients were pseudo-randomized by their baseline demographic factors using a propensity score-matching method. The PNE was based on a psychosocial model and began 1 week postoperatively in a group setting; five 1-h weekly sessions were conducted. The primary outcome was the walking pain score as measured by a numerical rating scale. The secondary outcomes were the pain catastrophizing scores as measured by the PCS, self-efficacy as measured by the Pain Self-Efficacy Questionnaire, and physical function. Measurements were taken at baseline (before surgery) and before discharge from the hospital (5 weeks postoperatively) to identify any intervention effects.

**Results:**

After propensity score matching, 52 pairs of patients were extracted. In the intervention group, 46 (88.5%) patients completed the PNE. In total, 44 patients in the intervention group and 52 patients in the control group were analyzed. Five weeks following surgery, the rehabilitation itself had also significantly decreased catastrophizing, and the difference between the two groups had only a small effect size (d = 0.44).

**Conclusions:**

These findings provide preliminary evidence that physical therapist-delivered PNE during hospitalization may help to at least slightly reduce pain catastrophizing in patients with catastrophizing prior to knee arthroplasty.

**Trial registration:**

This trial was retrospectively registered with ClinicalTrials.gov (UMIN000037114) on 19 June 2019.

## Background

Knee osteoarthritis (KOA) is a widespread chronic condition and one of the most common causes of musculoskeletal disability among all high-risk health problems [1]. The prevalence of KOA estimated by radiographic in Japan is 54.6% (42.0% male, 61.5% female) [2]. KOA is associated with disrupted sleep, depression, increased sedentary behavior, less physical activity, obesity, and polypharmacy, all of which decrease patients’ quality of life [3]. Therefore, because of the chronicity and complexity of KOA, effective treatment is necessary at all stages of the condition to manage affected patients’ quality of life.

Surgical treatments such as knee arthroplasty or high tibial osteotomy (HTO) for patients with severe KOA are commonly performed, and HTO is performed in younger patients than is knee arthroplasty [4]. A higher demand for surgical treatment by patients aged < 65 years is expected by the year 2030, which would represent a 17-fold growth rate from 2006 [5]. Therefore, the demand for HTO by young patients is expected to increase.

A study of 4400 patients who underwent knee arthroplasty showed that 35% had unexplained chronic pain 15 years following surgery [6]. Another study showed that 20% of patients had persistent function-limiting pain ≥6 months following knee arthroplasty despite an apparently normally functioning prosthesis [7]. Countermeasures against chronic pain are necessary after surgery for KOA.

The intensity of acute postoperative pain or the impairments caused by pain catastrophizing among patients who have undergone knee arthroplasty is associated with a risk of developing a chronic pain state and poor outcomes [8, 9]. Pain catastrophizing is the most consistent and powerful psychological predictor of acute postsurgical pain following knee arthroplasty [10]. Chan et al. [11] reported that despite usual postoperative care during hospitalization, many patients experienced significant pain, and they received inadequate information at discharge to effectively self-manage their postoperative knee pain. This indicates that non-pharmacologic therapy for KOA in patients with a high tendency to engage in postoperative pain catastrophizing requires education regarding the reduction of pain catastrophizing and information to manage pain.

In a comparison of the effects of patient education according to the contents of the education program, pain neuroscience education (PNE) provided a significantly better understanding of the neurophysiology of pain and a reduction of pain catastrophizing compared with pacing and self-management education [12]. Additionally, other systematic reviews and meta-analyses [13–15] showed that PNE appears to be effective in reducing pain, disability, and psychosocial factors; improving patient knowledge of pain; and minimizing healthcare utilization. However, PNE has not been proposed as a stand-alone treatment.

This study was performed to evaluate the effectiveness of a hospital-time PNE intervention combined with physical therapist-prescribed rehabilitation in patients undergoing HTO with respect to improvements in pain and pain-related psychological and physical function. Our hypothesis was that the intervention would result in an improvement in pain-related psychological factors at discharge (5 weeks postoperatively) compared with rehabilitation alone.

## Methods

### Study design

We performed this quasi-experimental study using data from two independent cohorts (one that received rehabilitation alone and one that received PNE combined with rehabilitation) at Fukuoka Rehabilitation Hospital, Nishi-ku, Fukuoka-shi Fukuoka, Japan from April 2014 to January 2018. The patients who underwent PNE combined with rehabilitation were recruited from 2016, and the patients who underwent rehabilitation alone were recruited from 2014 to 2016 retrospectively. We considered the possibility of bias in that the baseline characteristics (e.g., baseline demographics and other covariates) of the participants were more favorable than those of nonparticipants [16]. Thus, we applied propensity score matching to select a portion of nonparticipants with baseline characteristics similar to those of the participants and compared the treatment outcomes between the groups.

### Patients

The participants had already consented to undergo open-wedge HTO 2 to 4 weeks prior to their scheduled surgery.

Patients were eligible to participate if they (1) were able to read and speak Japanese and provided informed consent, (2) were ≥ 45 years old, (3) had a diagnosis of osteoarthritis as determined by their orthopedic surgeons, and (4) were scheduled for primary (not revision) unilateral open-wedge HTO.

Patients were excluded from the study if they (1) were scheduled for revision arthroplasty surgery; (2) were unable to or declined to provide consent for study participation; (3) had a self-reported diagnosis of inflammatory arthritis (i.e., rheumatoid arthritis, systemic lupus erythematosus, or ankylosing spondylitis); (4) had neurological or psychological disease; (5) were scheduled to undergo HTO because of a fracture, malignancy, or infection; (6) were scheduled for bilateral HTO; (7) were scheduled for unilateral arthroplasty; (8) reported plans to undergo hip or knee arthroplasty within 6 months after the current HTO; or (9) had a Pain Catastrophizing Scale (PCS) score of < 21. A previous study showed that patients with a PCS of < 21 had low catastrophization tendency [17].

### Sample size calculation

The minimal clinically important difference (MCID) of walking pain is reportedly ≥2 points when assessing pain using a numerical rating scale (NRS) [18]. We used a two-sided, two-group t-test of differences in means with alpha set at 0.05 and assumed that the intervention difference minus the control difference was an NRS score of ≥2 points for walking pain. The standard deviation used was 2.5 as shown in the pilot study [19]. We calculated that we needed a sample size of 33 in each group (total of 66) for a power of 80% to detect such a difference. A dropout rate of 30% was expected. In addition, analysis by the propensity score matching method may exclude a large number of patients; we assumed a 20% exclusion rate, and 99 patients in total were therefore recruited.

### Intervention and control groups

Patients in the intervention group (PNE combined with rehabilitation) underwent treatment from June 2016 to January 2018, and patients in the control group (rehabilitation alone) underwent treatment from April 2014 to May 2016.

### Rehabilitation

Patients in the control group underwent postoperative usual rehabilitation performed by physical therapists and occupational therapists. This was started the day after surgery and was performed six times a week. Full weight bearing on the operative side was not performed until 2 weeks after surgery, at which point patients bore weight according to their pain severity. Additionally, analgesic nonsteroidal anti-inflammatory drugs were taken three times a day from the day after surgery, and the dosage was decreased from 3 weeks postoperatively according to the pain severity.

### PNE

The biopsychosocial model-based PNE used in the present study was a physical therapist-led education program that comprised a lecture and practice and was developed based on the following factors described in previous studies [20–22]: physiological impairments (pain and muscle weakness), personal factors (knowledge, health beliefs, self-efficacy, and stress management), and behaviors during daily activities (avoidance of movement or exercise, eating and sleeping habits, and goal setting) (Table 1). Our PNE consisted of five individual sessions using a booklet that was designed to be completed during the hospitalization period. Each session was approximately 60 min in length. The first session was delivered approximately 1 week after the operation, and the subsequent four sessions were delivered within 5 weeks after the operation. The PNE was delivered by three physical therapists (two in session 1 only, one in sessions 2–5 only) with > 10 years of experience in treating patients undergoing HTO. The physical therapists who provided sessions 2 to 5 participated in a 2-day training program delivered by doctors, psychologists, and physical therapists specialized in cognitive behavioral therapy and pain management.

**Table 1:** Pain neuroscience education content

Session/time	Lecture	Practice
1. Purpose of the education	• Fear avoidance model	• Goal setting
1 week postoperatively	• Rehabilitation and education schedule	
2. Pain in knee osteoarthritis	• Biological psychology model	• Cognitive restructuring
2–5 weeks postoperatively^a^	• Descending inhibitory pathways	
3. Pain and sleep	• Sleep hygiene education	• Mindfulness
2–5 weeks postoperatively^a^	• Descending pain modulatory systems	• Distraction
4. Pain and lifestyle	• Pain and inactivity	• Activity pacing
2–5 weeks postoperatively^a^	• Effects of nutrition on inflammation	
5. Self-management	• Social cognitive theory	• Decisional balance
2–5 weeks postoperatively^a^	• Willpower and brain	

^a^Attend at random

The goal of the introduction to PNE was to have patients understand the states within the cycle of chronic pain induced by predisposing psychological factors, such as negative affectivity, negative appraisal, or anxiety sensitivity, in addition to the actual nociceptive pain associated with surgery. The patients were given an explanation regarding the transition of postoperative pain and the flow of rehabilitation, including PNE, to avoid increased anxiety regarding postoperative pain. Goal setting involved the development of explicit, reasonable, objective, and patient-centered goals.

The aim of the KOA-associated pain session was to provide patients with an understanding of how nerves are viewed as an alarm system that moves information from the tissues to the brain. This session explained the biology and physiology of nerves to the patients. A key element was to explain that pain may be a result of not only injured tissue but also (and likely more so) increased nerve sensitivity. The patients practiced cognitive restructuring, in which they brainstormed to identify and alter maladaptive thoughts and emotions related to their pain.

The aim of the pain and sleep session was to help patients understand that a decrease in descending inhibitory pathway pain by lack of pain knowledge, sleep, and medication adherence contributes to increased subjective pain sensitivity and increased spinal nociception. Mindful breathing and visual imaging were each practiced once as relaxation techniques.

The goal of the pain and lifestyle session was to provide patients with an understanding that a correct lifestyle greatly affects pain. Patients were given information on how nutrition affects inflammation and how pain and psychological disability influence sedentary behavior and moderate physical activity. Activity pacing involved strategies such as reducing the speed of activities, taking breaks, maintaining a consistent pace, and separating tasks into manageable components.

Finally, the aim of the self-management session was to help patients anticipate barriers to change and make plans to overcome those barriers. This session explained that successful long-term self-management requires motivation, and patients were lectured on social cognitive theory, transtheoretical models, and willpower based on neurophysiology and psychology. Decisional balance in this session involved weighing the perceived advantages and disadvantages of adherence to medication and diet management and exercise by brainstorming.

### Outcomes

The primary and secondary outcomes were valid and reliable self-reported measures of pain and pain-related psychological factors recommended by the European League Against Rheumatism [23]. These outcomes were evaluated before the operation (baseline) and 5 weeks after the operation (follow-up).

### Primary outcomes

The primary outcomes were pain at rest and while walking as measured with an NRS using terminal descriptors of “no pain” (score of 0) and “worst pain possible” (score of 10) [24, 25] and an MCID of 2.0 units [17].

### Secondary outcomes

The secondary outcomes were psychological factors and physical function.

Pain catastrophizing was measured using the PCS [26, 27] (score of 0–52, with higher scores indicating greater catastrophizing), and self-efficacy for pain was measured using the Pain Self-Efficacy Questionnaire (PSEQ) [28, 29] (score of 0–60, with higher scores indicating greater self-efficacy). Strength was assessed by measuring the isometric knee extension strength at 90° knee flexion in a sitting position using a dynamometer (μTas F-1; ANIMA Corporation, Tokyo, Japan). After a single, submaximal warm-up trial, the patients performed three trials of 5-s duration each, separated by 15 s of rest. The maximum force output (kgf) from the three trials (corrected for gravitational weight of the limb as appropriate) was recorded and converted to torque (Nm) by multiplying by and then normalizing to body mass (kgf/kg) [30]. Walking speed was assessed by the 10-m walking test, which was performed twice; in this test, the patients were instructed to walk at their preferred speed over a length of 15 m. Time was started at 2.5 m and stopped at 12.5 m, resulting in a steady-state measurement over 10 m [31]. Time was measured using a hand-held stopwatch, and the shortest time was used for the analysis. Demographic data comprised age and sex, health-related data comprised complications and body mass index, and disease-related data comprised the symptom duration, radiation severity, history of other knee operations, hospitalization season, and hospital stay.

### Statistical analysis

#### Propensity score matching

Propensity score matching entails the formation of matched sets of treated and untreated subjects who share similar propensity scores [32]. The most common implementation of propensity score matching is one-to-one or pair matching, in which pairs of treated and untreated subjects are formed such that matched subjects have similar propensity scores [33]. In this study, propensity scores were calculated using age, sex, complications, body mass index, symptom duration, and radiation severity as preoperative factors. The one-to-one nearest-neighbor matching method was applied; in this method, control patients with propensity scores most closely approximating those of patients in the intervention group were selected.

### Outcome analysis

Analyses were performed using SPSS version 25.0 (IBM Corp., Armonk, NY, USA). Two-sided hypothesis tests were used, and a *P* value of < 0.05 was considered statistically significant. A modified intention-to-treat analysis was performed to define all participants who had baseline measurements and underwent at least one PNE session. The missing baseline data estimates from 25 imputed data sets were combined using Rubin’s rules [34]. Descriptive statistics were used to describe the baseline characteristics of the patients in each group. All data were tested for normality using the Shapiro–Wilk W test. Student’s t test or the Mann–Whitney U test (for continuous variables) and the χ^2^ test or Fisher’s exact test (for categorical variables) were applied to identify any baseline differences between the groups. Within-group differences from baseline at each follow-up time point were summarized using the adjusted mean change, and 95% confidence intervals were examined for each variable using analysis of covariance measurements adjusted for baseline scores. Cohen’s d effect size to describe the magnitude of the treatment effect was as follows: small, 0.20 to < 0.50; medium, 0.50 to < 0.80; and large, ≥0.80 [35].

The proportion of patients in each group who attained the MCID for the primary outcomes (reduction of 2.0 units for pain) was calculated. Based on the perceived global change overall and in pain, patients who reported a > 2.0-unit reduction of the MCID were classified as improved, and those who reported a lesser reduction were classified as not improved. Differences within both groups at baseline and follow-up were evaluated with the χ^2^ test, and relative risk was analyzed. A sensitivity analysis was performed and included patients who did not participate in PNE.

## Results

### Characteristics of the study sample

**Fig. 1:**
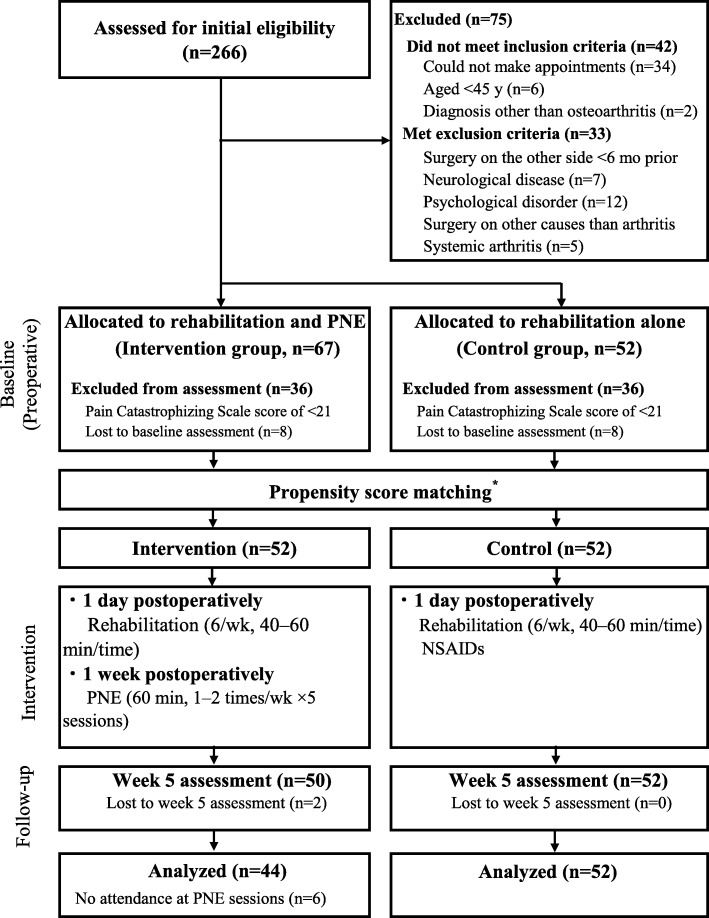
Study Protocol

Figure 1 shows the study flowchart. Of the 266 screened patients who underwent HTO, 197 (74.0%) patients were eligible and agreed to enroll in this study. Of these 197 patients, 119 (60.4%) had a PCS score of ≥21 and thus qualified for the current study. These patients were then pseudo-randomized into each group by a propensity score-matching analysis, resulting in 52 patients in the intervention group and 52 patients in the control group. Eight patients in the intervention group were excluded from the analysis (two because of > 50% data loss by the week 5 assessment, and six because of lack of attendance at the PNE sessions during the study period). Table 2 shows the baseline demographics and knee symptom characteristics of the patients in the intervention and control groups before and after the propensity score-matching analysis. There were significant differences in age and complications before study enrollment between the two groups, but no significant differences were present after adjustment.

**Table 2:** Baseline descriptive characteristics before and after propensity score matching

	Control		Intervention					
			Unadjusted			Adjusted		
	(*n* = 52)		(*n* = 67)		*P* value	(*n* = 52)		*P* value
Mean (SD) age, years	63.7	(8.6)	67.0	(8.3)	0.042	66.2	(8.0)	0.146
Female, n (%)	32	(67)	46	(75)	0.393	37	(77)	0.364
Mean (SD) height, cm	158.4	(9.2)	157.4	(7.4)	0.549	157.4	(6.5)	0.552
Mean (SD) weight, kg	65.9	(13.1)	61.7	(10.7)	0.069	62.0	(11.0)	0.117
Mean (SD) BMI, kg/m2	26.1	(3.9)	24.8	(3.4)	0.072	24.9	(3.6)	0.127
Symptom duration, n (%)								
< 3 mo	36	(69)	43	(64.2)	0.415	33	(63.5)	0.678
3–6 mo	4	(8)	3	(4.5)		3	(5.8)	
6–1 y	4	(8)	3	(4.5)		3	(5.8)	
> 1 y	8	(15)	18	(26.9)		13	(25.0)	
Complications, n (%)								
Diabetes	15	(29)	17	(25)	0.683	9	(17)	0.244
Hypertension	28	(54)	29	(43)	0.272	24	(46)	0.557
Varicose veins	10	(19)	25	(37)	0.042	17	(33)	0.179
Radiation severity, n (%)								
Operative side								
Kellgren–Lawrence grade					0.113			0.148
II	11	(21)	26	(39)		20	(38.5)	
III	27	(52)	25	(37)		20	(38.5)	
IV IV	14	(27)	16	(24)		12	(23)	
Nonoperative side Nonoperative side					0.662			0.563
Kellgren–Lawrence grade Kellgren–Lawrence grade								
Non-KOA	31	(59.6)	33	(49.3)		26 (50)		
II	2	(3.8)	2	(3.0)		1 (2)		
III	2	(3.8)	7	(10.4)		4 (8)		
IV	1	(1.9)	2	(3.0)		0 (0)		
Postoperatively	16	(30.8)	23	(34.3)		21 (40)		
Hospitalization month, n (%)								
3–5	16	(31)	12	(18)	0.311	11	(21)	0.587
6–8	12	(23)	24	(36)		17	(33)	
9–11	16	(31)	21	(31)		17	(33)	
12–2	8	(15)	10	(15)		7	(13)	

*SD* standard deviation, *BMI* body mass index, *KOA* knee osteoarthritis

### Comparison between intervention group and control group

The PNE intervention time was 4.2 ± 1.0 h. The length of stay in the surgery ward was not significantly different between the intervention group (42 [36–48] days) and control group (39 [36–45] days). Table 3 summarizes the results of the before-and-after comparison. The NRS and PCS scores showed significant improvement in both groups. Additionally, the intervention group showed significantly greater improvement in the PSEQ score and knee extension strength (nonoperative side) among the secondary outcomes, but showed no significant differences in the other secondary outcomes. Table 4 shows the changes between the groups. The differences in the baseline-adjusted discharge NRS pain scores at rest and walking between the two groups were as follows. The intervention group showed a mean improvement of − 1.0 (standard deviation [SD] = 0.9) and − 2.1 (SD = 1.6) points, while patients in the control group showed a mean improvement of − 1.1 (SD = 1.0) and − 1.9 (SD = 1.7) points. These differences were not significant at *p* = 0.47 and 0.46, respectively. Additionally, a significant proportion of participants in both groups did not exceed the MCID at both time points (Table 5). The intervention group showed a mean improvement in the PCS score of 13.7 (SD = 8.6) points, while the control group showed a mean improvement of 9.9 (SD = 8.7) points. This difference of 3.8 PCS points between the two groups was significant at *p* = 0.036 (d = 0.44). However, the PSEQ score and motor function among the secondary outcomes showed no significant differences between the two groups. In addition, the effectiveness of the PCS and PSEQ decreased in the sensitivity analysis (Table 6).

**Table 3:** Comparison of baseline and follow-up outcomes in each group

Outcomes	(n)	Intervention		*P* value	(n)	Control		*P* value
		Baseline	Follow-up			Baseline	Follow-up	
Primary (NRS score; 0–10)								
Pain at rest	**44**	2.0 (2.6)	1.1 (1.3)	0.033	**52**	1.9 (2.3)	0.8 (1.2)	0.001
Pain during walking	**42**	4.7 (2.3)	2.2 (1.4)	< 0.001	**47**	4.2 (2.8)	2.6 (2.1)	0.003
Secondary								
PCS score (0–52)	**42**	30.3 (6.5)	16.9 (9.7)	< 0.001	**48**	30.8 (7.7)	20.7 (8.4)	< 0.001
PSEQ score (0–60)	**42**	37.6 (10.6)	43.4 (10.2)	0.005	**48**	36.3 (11.1)	38.7 (12.8)	0.215
Knee extension (kgf/kg)								
Operative side	**37**	0.25 (0.09)	0.22 (0.10)	0.071	**48**	0.30 (0.14)	0.30 (0.13)	0.827
Nonoperative side	**37**	0.31 (0.09)	0.35 (0.11)	0.015	**44**	0.30 (0.14)	0.33 (0.14)	0.126
10-m walking test (s)	**35**	8.9 (3.2)	9.6 (2.4)	0.186	**47**	9.1 (3.3)	10.5 (7.6)	0.135

Data are presented as mean (standard deviation). Baseline: preoperative, Follow-up: 5 weeks postoperatively, *NRS* numerical rating scale, *PCS* Pain Catastrophizing Scale, *PSEQ* Pain Self-Efficacy Questionnaire

**Table 4:** Comparison of baseline and follow-up outcomes between groups^a^

	Follow-up to baseline		Difference in change between groups^a^		
	Intervention	Control	Mean (95% CI)	*P* value	Cohen’s d
Primary					
Pain at rest (NRS)	−1.0 (0.9)	− 1.1 (1.0)	0.1 (−0.2, 0.4)	0.470	0.07
Pain during walking (NRS)	−2.1 (1.6)	−1.9 (1.7)	−0.2 (− 0.8, 0.4)	0.460	0.14
Secondary					
Pain catastrophizing (PCS)	−13.7 (8.6)	−9.9 (8.7)	−3.8 (−0.2, −7.4)	0.036	0.44
Self-efficacy (PSEQ)	6.0 (10.3)	2.1 (10.5)	4.0 (−0.3, 8.3)	0.070	0.38
Knee extension					
Operative side	−0.03 (0.14)	0.00 (0.13)	−0.03 (− 0.08, 0.03)	0.350	0.19
Nonoperative side	0.05 (0.11)	0.02 (0.12)	0.03 (−0.02, 0.07)	0.248	0.24
10-m walking test	0.1 (5.5)	1.8 (5.0)	−1.7 (−3.8, 0.5)	0.121	0.32

Data in first two columns are presented as mean (standard deviation). Baseline: preoperative, Follow-up: 5 weeks postoperatively, *95% CI* 95% confidence interval, *NRS* numerical rating scale, *PCS* Pain Catastrophizing Scale, *PSEQ* Pain Self-Efficacy Questionnaire. ^a^Values adjusted for baseline scores using analysis of covariance

**Table 5:** MCID of primary outcome

	n (%)		*P* value	RR (95% CI)
	Intervention	Control		
MCID for pain at rest (NRS)				
2.0 units of improvement				
Yes	16 (36)	18 (35)	0.18	1.03 (0.76, 1.34)
No	28 (64)	34 (65)		
MCID for pain during walking (NRS)				
2.0 units of improvement				
Yes	25 (60)	24 (51)	0.35	1.21 (0.76, 1.93)
No	17 (40)	23 (49)		

*MCID* minimal clinically important difference, *RR* relative risk, *95% CI*: 95% confidence interval, *NRS* numerical rating scale

**Table 6:** Comparison of baseline and follow-up outcomes between the two groups by intention-to-treat analysis^a^

	Follow-up to baseline		Difference in change between groups		
	Intervention	Control	Mean (95% CI)	*P* value	Cohen’s d
Primary					
Rest (NRS)^b^	−0.9 (1.0)	−1.0 (1.0)	0.1 (−0.2, 0.4)	0.65 0.11	
Pain during walking (NRS)^c^	−2.0 (1.7)	−1.8 (1.7)	−0.2 (− 0.9, 0.4)	0.47 0.14	
Secondary					
Pain catastrophizing (PCS)^d^	−13.4 (8.7)	−10.0 (8.6)	−3.5 (−6.9, 0.0)	0.050 0.40	
Self-efficacy (PSEQ)^e^	4.8 (10.7)	2.0 (10.9)	2.8 (−1.5, 7.1)	0.20 0.26	
Quadriceps muscle strength					
Operative side^f^	−0.03 (0.14)	0.00 (0.12)	−0.03 (− 0.08, 0.03)	0.35 0.23	
Nonoperative side^g^	0.05 (0.11)	0.02 (0.11)	0.03 (−0.02, 0.07)	0.25 0.27	
10-m walking test^h^	0.3 (5.1)	1.8 (4.8)	−1.5 (−3.5, 0.5)	0.147 0.3	

Data in first two columns are presented as mean (standard deviation). Baseline: preoperative, Follow-up: 5 weeks postoperatively, *PNE* pain neuroscience education, Reha: rehabilitation, 95% CI: 95% confidence level, *NRS* numerical rating scale, *PCS* Pain Catastrophizing Scale, *PSEQ* Pain Self-Efficacy Questionnaire

^a^Values adjusted for baseline scores using analysis of covariance

^b^Intervention (*n* = 50), Control (*n* = 52)

^c^Intervention (*n* = 48), Control (*n* = 47)

^d^Intervention (*n* = 49), Control (*n* = 48), ^e^Intervention (*n* = 48), Control (*n* = 48)

^f^Intervention (*n* = 43), Control (n = 48)

^g^Intervention (*n* = 43), Control (*n* = 44)

^h^Intervention (*n* = 41), Control (*n* = 47)

## Discussion

Five weeks of postoperative education on the neuroscience of pain along with rehabilitation improved patients’ pain catastrophizing compared with patients who underwent rehabilitation only. To the best of our knowledge, this is the first study to show that a combination of postoperative rehabilitation and PNE can contribute to improvements in patients’ catastrophizing, which is a risk factor for chronic pain after HTO.

Intervention studies using the cut-off PCS have not been unified at > 16 points [36] or > 23 points [37]. A PCS of > 16 points affects patients’ long-term postoperative outcomes [38]. Therefore, a study of patients with a PCS of > 16 points is necessary. A large-scale study involving 2854 patients showed that the lower quartile of the PCS of was < 21 points [17]. The cut-off in the present study was 21 points.

The short-term effects of the addition of PNE to physiotherapy interventions on pain have been observed in patients with chronic pain [39] but not in patients with acute pain after surgery [40, 41]. Our results suggest that both groups improved by the same degree, with no significant difference between the groups; this supports the results of patients with acute pain. In a systematic review and meta-analysis of analgesic interventions for postoperative acute pain, nonsteroidal anti-inflammatory drugs demonstrated significant analgesic effects [42]. Therefore, the effect of PNE on short-term postoperative acute pain may be greatly influenced by the analgesic effect of nonsteroidal anti-inflammatory drugs used after surgery.

The mean difference in the PCS score between the two groups was 3.8 points, which is a small effect size. Lluch et al. [41] reported that preoperative PNE combined with knee joint mobilization produced benefits for pain catastrophizing compared with biomedical education combined with knee joint mobilization. This effect size was similar to our study, suggesting that the timing of the education intervention did not influence these effects. Additionally, the pre- and post-test mean improvements in the PCS score in the intervention and control groups were substantially larger than the minimal clinical difference of approximately 9.1 points [43]. Pain catastrophizing can be substantially reduced through a range of interventions such as surgery, physiotherapy, and even pharmacotherapy [42]. However, pain coping skills training before and after knee arthroplasty resulted in a large effect size with a difference of 10.3 PCS between the groups [44]. Therefore, more effective PNE may require additional preoperative intervention. Additional benefits of PNE that can help to improve psychological factors include the potential to prevent high rates of opioid prescription [45] and lower medical expenses in the long term [46]. In the present study, however, verification of these factors was not performed. It will be necessary to verify these factors in future studies. The PSEQ scores did not change significantly between the groups. In previous studies, improvement of PSEQ scores suggested that activity pacing and interviews for enhancing motivation should be incorporated into clinical practice as effective therapeutic interventions [47, 48]. The interventions in the present study provided an overview of active pacing to the study group. The patients then brainstormed ways to change their pain when not maintaining a consistent pace, such as slowing down activities or taking breaks. However, individual interviews were not conducted. The results of this study may have been affected by the lack of individual interventions.

The present study showed no significant difference in the strength or walking ability of the operative knee between the intervention group and the control group. Louw et al. [49] and Beaupre et al. [50] reported that a preoperative education program resulted in no differences in postoperative walking ability or knee strength. These results support the results of the present study. One interesting point that was not statistically examined and that would require a post-hoc analysis was the apparent a difference between the surgical and nonsurgical legs between the groups. The rehabilitation-only group showed no change in the operative side and a slight increase in the nonoperative side, whereas the PNE + rehabilitation group showed loss of strength on the operative side and increased strength on the nonoperative side. This may warrant discussion and suggestion for further investigation.

Our study has some limitations. First, quasi-experimental designs are viable alternatives to randomized clinical trials. The patients in our study were not randomly assigned to the two treatment conditions, and we cannot be certain whether the differences were due to the intervention or to pretreatment differences between the groups. For example, we were unable to control all potential confounders such as physical activity, educational history, and economic aspects. Second, history is another internal validity threat to quasi-experimental designs. Our control group was treated 1 to 2 years prior to the patients who received PNE. Although the surgical and implantation procedures may have varied throughout the study period, we are unaware of evidence indicating that the differences found in this study were due to differences in surgical techniques over this relatively short time interval. This aspect should be taken into account when interpreting the results. Third, the patients were not blinded to the treatments, which could have resulted in overestimation of the benefits. The PCS score that proved effective in this study was based on a questionnaire survey, and the possibility of describing good results after the intervention in the PNE group cannot be denied. Fourth, the differences in the effect of PNE might have been only due to an increase in contact hours with the patients. Fifth, this study included a mixture of highly catastrophizing and non-catastrophizing patients. High catastrophizing is generally accepted to be present in patients with a PCS score of > 30 points [51]. In the future, we will assess highly catastrophizing patients with a PCS cut-off of > 30 points.

## Conclusion

Physical therapist-prescribed rehabilitation combined with postoperative PNE, consistent with a biopsychosocial approach in patients undergoing HTO, conferred small benefits in pain catastrophizing compared with rehabilitation alone. Further studies will need to provide PNE intervention timing and long-term verification that PNE prevents high rates of opioid prescription and lowers health care costs.

## Data Availability

The dataset supporting the conclusions of this article is proprietary to Fukuoka Rehabilitation Hospital / Fukuoka Reha Orthopedic Clinic and will not be shared because the hospital restricts sharing of the raw data with concerned personnel only.

## Abbreviations

HTO: Higher tibial osteotomy
KOA: Knee osteoarthritis
MCID: Minimal clinically important difference
NRS: Numerical rating scale
PCS: Pain Catastrophizing Scale
PNE: Pain neuroscience education
PSEQ: Pain Self-Efficacy Questionnaire
SD: Standard deviation
